# Ex vivo cultivated oral mucosal epithelial cell transplantation for limbal stem cell deficiency: a review

**DOI:** 10.1186/s13287-020-01783-8

**Published:** 2020-07-21

**Authors:** Joao Victor Cabral, Catherine Joan Jackson, Tor Paaske Utheim, Katerina Jirsova

**Affiliations:** 1 Laboratory of the Biology and Pathology of the Eye, Institute of Biology and Medical Genetics, First Faculty of Medicine, Charles University and General University Hospital in Prague, Prague, Czech Republic; 2 Department of Medical Biochemistry, Oslo University Hospital, Oslo, Norway; 3 Department of Oral Biology, Faculty of Dentistry, University of Oslo, Oslo, Norway; 4 Department of Plastic and Reconstructive Surgery, Oslo University Hospital, Oslo, Norway; 5 Department of Ophthalmology, Sørlandet Hospital Trust Arendal, Arendal, Norway

**Keywords:** Cultivated oral mucosal epithelial cell, Limbal stem cell deficiency, Oral mucosal epithelial cells, Tissue regeneration

## Abstract

Destruction or dysfunction of limbal epithelial stem cells (LESCs) leads to unilateral or bilateral limbal stem cell deficiency (LSCD). Fifteen years have passed since the first transplantation of ex vivo cultivated oral mucosal epithelial cells (COMET) in humans in 2004, which represents the first use of a cultured non-limbal autologous cell type to treat bilateral LSCD. This review summarizes clinical outcomes from COMET studies published from 2004 to 2019 and reviews results with emphasis on the culture methods by which grafted cell sheets were prepared.

## Background

Damage to the limbus can lead to a decrease in limbal epithelial stem cells (LESCs) and dysfunctional homeostasis of the corneal epithelium. This failure, termed limbal stem cell deficiency (LSCD) [1–3], leads to disruption of the barrier function and invasion of conjunctival cells onto the corneal surface [4, 5]. Conjunctivalization is followed by vascularization, chronic inflammation, photophobia, recurrent pain, and decreased vision [4, 6–8]. LSCD is classified as partial or total and may occur unilaterally or bilaterally [9].

Conjunctival limbal autograft (CLAU) and cultivated limbal epithelium transplantation (CLET) are procedures often used in the treatment of unilateral LSCD [10, 11]. However, patients with bilateral total LSCD do not have limbal tissue available for use in either CLAU or CLET. Thus, options for a source of LESCs are limited to living-related or cadaveric donors and entail use of immunosuppression to prevent rejection [12].

In 2004, Nakamura and co-workers performed the first transplantation of autologous oral epithelial cells cultured ex vivo on human amniotic membrane (AM) to offer an alternative to use of allogenic tissue and avoid immunosuppression [13]. The treatment of LSCD using ex vivo cultivated oral mucosal epithelial cell transplantation (COMET) minimizes the risk of graft rejection and has the added advantage that it can be repeated if necessary. However, neo-angiogenesis following transplantation is a drawback associated with this procedure [13]. This review summarizes clinical outcomes from COMET case series from 2004 to 2019 and reviews the methods used in preparation of transplanted cell sheets.

### General analysis of studies

The review was prepared by searching the Ovid MEDLINE database using search terms: limbus corneae, limbus, limbal stem cell deficiency, corneal epithelium, cornea, mouth mucosa, and transplantation. We found 24 studies published over the past fifteen years [13–36]. A case report of one patient (one eye) was excluded from this review [37].

COMET has been performed in Japan [13–19, 23–25, 27, 29, 30], Taiwan [20, 21, 28], India [22], France [26], the UK [31], Poland [32], Thailand [33], Iran [34], South Korea [35], and China [36]. In total, 343 eyes of 315 patients (64% men and 36% women) were included. The age range was from eight to 86 years, the mean age was 46.5 (± 18.6) and 50.8 (± 21.5) years for males and females, respectively. About 26% of male and 23% of female patients were younger than 30 years, while about 28% and approximately 45%, respectively, were older than 60 years.

Three hundred and twenty LSCD eyes were classified as totally deficient, eight eyes as partial [32, 33]. One study classified all 5 eyes as severe LSCD [21]. Nine studies included patients with bilateral LSCD [13–16, 19, 26, 31, 33, 34], two studies included both bilateral and unilateral cases [22, 30], and one study enrolled only patients with unilateral LSCD [25].

### Patients and surgery

#### Etiology

**Fig. 1:**
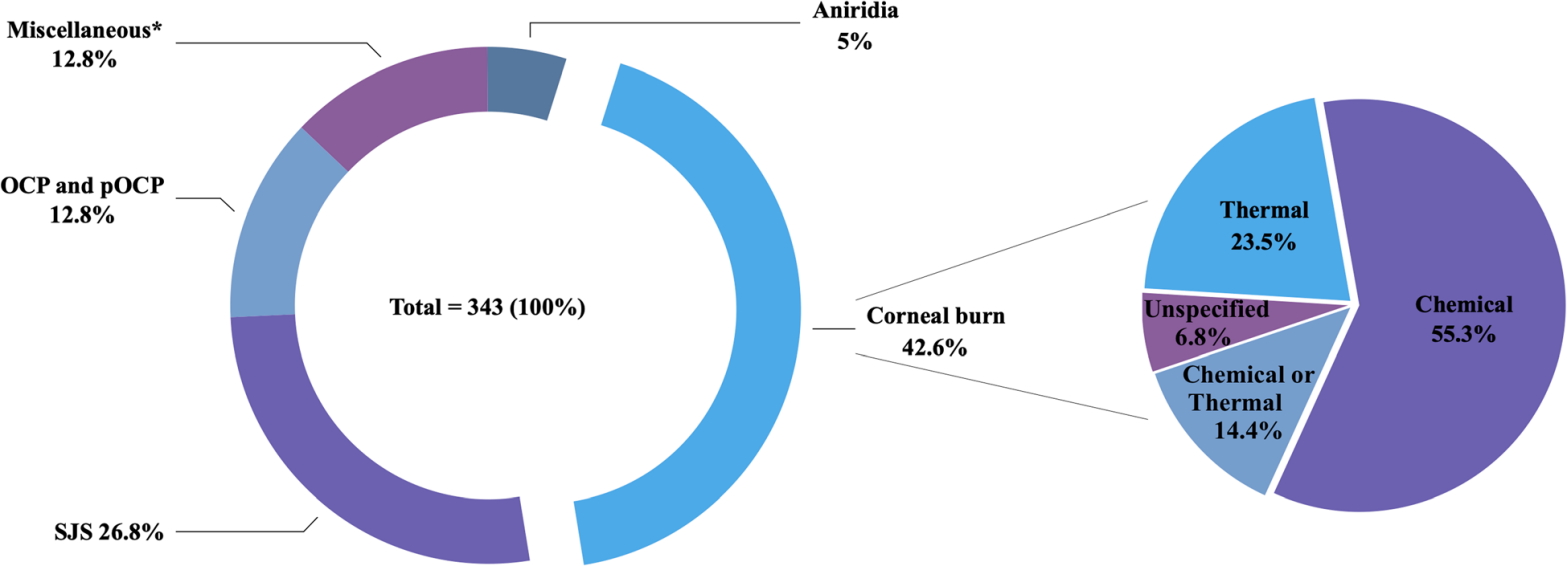
Etiology of limbal stem cell deficiency (LSCD). Percentages are according to the number of eyes. OCP, ocular cicatricial pemphigoid; pOCP, pseudo-ocular cicatricial pemphigoid; SJS, Stevens-Johnson syndrome. *Miscellaneous (%): trachoma (1.45), post keratitis (1.45), idiopathic (1.2), Lyell syndrome (1.2), rosacea keratitis (0.9), congenital aniridia (0.6), contact lens hypoxia + congenital aniridia (0.6), neuroparalytic keratitis (0.6), Behcet’s disease (0.6), graft-versus-host disease (0.6), squamous cell carcinoma (0.6), gelatinous drop-like dystrophy (0.3), multiple eye surgery (0.3), advanced pterygium (0.3), ocular trauma (0.3), contact lens hypoxia (0.3), cystinosis (0.3), severe Groenouw dystrophy (0.3), hepatitis C (0.3), radiation keratopathy (0.3), Salzmann’s corneal degeneration (0.3), and drug toxicity (0.3)

The most common etiology of LSCD is corneal burn (146/343 eyes; 42.6%) resulting from chemical, thermal or unspecified causes, followed by Stevens-Johnson syndrome (SJS) (92/343 eyes; 26.8%) (Fig. 1 and Table 1). Ocular cicatricial pemphigoid (OCP) and pseudo-ocular cicatricial pemphigoid (pOCP) together composed the third most common cause of LSCD receiving COMET (44/343 eyes; 12.8%).

**Table 1:** Summary of clinical studies

Author, year	Etiology	No. of eyes/No. of patients	Dry eye assessment pre-operatively
Nakamura et al., 2004 [13]	SJS × 3, chemical burns × 3	6/4	Yes
Nishida et al., 2004 [14]	SJS × 1, OCP × 3	4/4	Yes
Inatomi et al., 2006 a [15]	SJS × 7, chemical injury × 5, thermal injury × 1, pOCP × 1, idiopathic × 1	15/12	Yes
Inatomi et al., 2006 b [16]	SJS × 1, chemical injury × 1	2/2	Yes
Ang et al., 2006 [17]	SJS × 7, thermal injury × 1, chemical injury × 1, OCP × 1	10/10	Yes
Nakamura et al., 2007 [18]	SJS × 3, chemical injury × 3	6/5	NA
Satake et al., 2008 [19]	SJS × 2, pOCP × 2	4/4	NA
Chen et al., 2009 [20]	Chemical burn × 3, thermal burn × 1	4/4	NA
Ma et al., 2009 [21]	Chemical burn × 3, thermal burn × 2	5/5	NA
Priya et al., 2011 [22]	SJS × 1, chemical injury × 9	10/10	Yes
Satake et al., 2011 [23]	SJS × 12, chemical or thermal injury × 11, OCP × 9, pOCP × 7, gelatinous drop-like dystrophy × 1	40/36	Yes
Nakamura et al., 2011 [24]	SJS × 11, chemical or thermal injury × 1, OCP × 4, squamous cell carcinoma × 2, graft-versus-host disease × 1	19/17	Yes
Takeda et al., 2011 [25]	Chemical burn × 1, thermal burn × 2	3/3	NA
Burillon et al., 2012 [26]	Corneal burn × 9, neuroparalytic keratitis × 2, rosacea keratitis × 3, Lyell syndrome × 4, severe trachoma × 1, contact lens hypoxia × 1, congenital aniridia × 1, cystinosis × 1, severe Groenouw dystrophy × 1, hepatitis C × 1, contact lens hypoxia + congenital aniridia × 2	26/25	Unclear^a^
Hirayama et al., 2012 [27]	Chemical injury × 12, pOCP × 12 (trachoma × 4, Behcet’s disease × 2, thermal burn × 1 and post keratitis × 5), SJS × 4, OCP × 4	32/32	Partially (27/32)
Chen et al., 2012 [28]	Chemical burn × 4, thermal burn × 2	6/6	NA
Sotozono et al., 2013 [29]	SJS × 21, OCP × 10, chemical or thermal injury × 7, idiopathic × 3, radiation keratopathy × 1, graft-versus-host disease × 1, congenital aniridia × 1, Salzmann’s corneal degeneration × 1, drug toxicity × 1	46/40	Unclear^a^
Sotozono et al., 2014 [30]	SJS × 3, thermal injury × 3, chemical injury × 2, OCP × 2	10/9	Unclear^a^
Kolli et al., 2014 [31]	Chemical burn × 2	2/2	Partially (1/2)
Dobrowolski et al., 2015 [32]	Aniridia × 17	17/13	NA
Prabhasawat et al., 2016 [33]	SJS × 10, chemical burn × 7, multiple eye surgery × 1, advanced pterygium × 1, ocular trauma × 1	20/18	Yes
Baradaren-Rafii et al., 2017 [34]	Chemical burn × 14	14/14	Yes
Kim et al., 2018 [35]	SJS × 6, OCP × 1, chemical burn × 1	8/8	NA
Wang et al., 2019 [36]	Chemical injury × 16, thermal injury × 18	34/32	NA

*LSCD* limbal stem cell deficiency, *NA* not available, *OCP* ocular cicatricial pemphigoid, *pOCP* pseudo-ocular cicatricial pemphigoid, *SJS* Stevens-Johnson syndrome

^a^These studies mentioned that dry eye patients received artificial tears in the post-operative management, but it was not stated whether dry eye was assessed in all patients

#### Diagnosis

Diagnosis of LSCD is based on the following clinical features: irregular corneal surface with loss of light reflex, corneal epithelial opacity, loss of limbal palisades of Vogt, fluorescein staining, epithelial thinning in a vortex pattern, corneal neovascularization, peripheral pannus, persistent epithelial defect (PED), corneal stroma scarring, and opacification [6, 38].

Corneal conjunctivalization can be confirmed clinically using in vivo confocal microscopy (IVCM) to define the phenotype of cells on the cornea (conjunctival epithelial cells are hyperreflective with bright nuclei and ill-defined borders, whereas corneal epithelial cells are well-defined with bright borders and dark cytoplasm) [39]. Conjunctival tissue contains goblet cells (GCs) and blood vessels, which can also be seen using IVCM [39]. Impression cytology (IC) is another method used to detect GCs on the corneal surface [4]. In case of GC absence due to severe ocular surface damage, conjunctival (but not corneal) mucins (mucin 1) [40] or keratins (keratin 7, -13, and -15) can be detected using immunocytochemistry [41–43]. Clinical features were used in diagnosis of LSCD in 18/24 studies [13–16, 19, 22–27, 29–33, 35, 36], five of these studies also used IC (Table 1) [19, 23, 31, 33, 36].

#### Pre-operative considerations

Some studies reported previous surgeries, including AM transplantation (38 eyes) [13, 15, 20–22, 28, 30, 35], and penetrating keratoplasty (PKP) (8 eyes) [14–16, 20, 21, 34, 35], or other (57 eyes) [29, 36]. Moreover, 21 eyes had previously undergone CLAU or allograft transplantation [13–15, 18–22, 35]. In total, 148 earlier surgeries were reported. Thus, the number of eyes previously treated was 119, more than a third (34.7%) of the total number of eyes included in this review [13–16, 18–22, 28–30, 34–36].

#### Prognostic factors

The presence of pre-operative epithelial defects and/or poor tear production may affect successful outcome [23, 44, 45]. Thus, numerous studies included assessment of dry eye in the pre-operative evaluation (Table 1) [13–17, 22–24, 27, 31, 33, 34]. DeSousa et al. recommend that adnexal abnormalities, including the health and function of the eyelids, fornix, and tear film, be assessed and improved prior to surgery to ensure the best chance of epithelial healing [46]. Conjunctival swab has revealed the presence of pathogenic organisms, which is likely due to a poor ocular surface and absence of a tear film. Therefore, performing a conjunctival swab culture before COMET to ensure a receptive ocular surface is suggested [47]. A complete oral exam is also recommended as successful culture of oral mucosal epithelial cells (OMECs) sheets may be affected by poor oral hygiene and smoking [15, 34].

#### Surgery

Surgical technique was similar in all studies. First, the conjunctival tissue was removed from the corneal surface, up to 3 mm away from the limbus to expose the corneal stroma [14, 17, 26]. Dissection of symblepharon was performed where necessary, and in some cases, AM was grafted onto the bare sclera to reconstruct the conjunctival fornix [17, 29, 30, 35]. In several cases, the subconjunctival space was treated with Mitomycin C [13, 15–17, 19, 22, 24, 27, 33]. A cultured OMEC sheet measuring from 14 [32] to 23.4 mm [14] diameter was transferred onto the corneal surface. Most of the studies used sutures to secure the graft in place [13, 15–34, 36]. Sutures were not used if the cell sheet was carrier-free [14, 26, 27, 35]. A study also used tissue adhesive glue [33], one used fibrin glue plus temporary tarsorrhaphy [35], and another used lateral tarsorrhaphy [34].

After surgery, AM [31] or therapeutic contact lenses (CLs) [13–36] were typically applied for 1 month [20, 29, 30] or for up to 3 months [24, 36] to protect the graft. One study reported adverse events attributed to hypoxia caused by extended use of CLs [26].

#### Post-operative considerations

Post-operative management varied considerably across the studies. A moist ocular surface post-COMET has been shown to be an important criterion for success [14, 24]. This was achieved by frequent application of preservative-free artificial tears [14, 19, 23, 26, 27, 32–34, 36], autologous serum eye drops [19, 21, 23, 31–33, 35, 36], or water-retaining hyaluronic acid [19, 23, 35]. One study occluded the lacrimal punctum to increase tear retention [14]. Topical antibiotics were applied in all studies, generally from 2 weeks [32, 33] up to 6 months [22]. Post-operative inflammation was controlled by topical steroids alone [27, 31–33] or in combination with systemic steroids [13, 14, 17, 19, 21–24, 26, 29, 30, 34–36]. The length of the treatment varied from 1 week [14, 26] up to 2 months [13, 21, 34]. Two studies tapered the dose-dependent on the patient response [29, 30]. In some studies, immunosuppression in the form of cyclosporine [17, 24, 29, 30] or cyclophosphamide [13, 21] was used to control post-operative inflammation, and topical tacrolimus [34] was used to decrease the risk of allograft rejection following PKP.

### Characteristics of the culture protocol used in clinical studies

#### Biopsy

The smallest tissue sample was ~ 4.7 mm^2^, obtained by using a 3-mm diameter biopsy punch [31], the largest ranged from 120 to 200 mm^2^ (Table 2) [35]. Fourteen studies used tissue from the buccal mucosa [14, 19, 21–23, 26–33, 36], and two from the lip [34, 35].

**Table 2:** Size and location of oral mucosal biopsy used in COMET

Studies	Biopsy size (mm^**2**^)	Location of biopsy
[13]	2–3	NA
[14]	9	Buccal mucosa
[15]	2–3	NA
[16]	3–5	NA
[17]	2–3	NA
[18]	NA	NA
[19]	50.24^a^	Buccal mucosa (inferior)
[20]	36	NA
[21]	36	Buccal mucosa
[22]	8	Buccal mucosa
[23]	50.24^a^	Buccal mucosa (inferior)
[24]	NA	NA
[25]	NA^c^	NA
[26]	9	Buccal mucosa (cheek)
[27]	50.24^a^	Inferior buccal mucosa
[28]	36	Buccal mucosa
[29]	9.42^b^	Buccal mucosa
[30]	9.42^b^	Buccal mucosa
[31]	4.71^c^	Buccal mucosa (cheek, 20 mm behind the angle of the mouth)
[32]	3–5	Buccal mucosa (inferior)
[33]	100	Buccal mucosa
[34]	NA	Labial mucosa (behind the lip)
[35]	120–200	Labial mucosa (inside the inferior lip)
[36]	16	Buccal mucosa (cheek)

*NA* not available

Area of an ^a^8-, ^b^6-, or ^c^3-mm diameter biopsy punch

#### Culture methods

Cell suspension was the most common culture system (23 studies), in which single OMECs were released from tissue using enzymatic treatment (Table 3) [13–30, 32–36]. All but one [33] of the cell suspension cultures reported standard use of 3T3 mouse fibroblasts in coculture, as a feeder layer [13–30, 32, 34–36]. The explant method was investigated in one study, where culture of the biopsy on AM demonstrated faster growth compared to culture on a feeder layer [31]. In vitro work has also shown that OMEC sheets maintain a comparable epithelial stem cell phenotype when cultured on autologous dermal fibroblasts compared with use of 3T3 mouse fibroblasts [48]. Culture time was typically 2 to 3 weeks; the shortest was 1 week [32]. Good manufacturing practice (GMP) regulations were followed in four studies from Japan [29, 30], South Korea [35], and the UK [31].

**Table 3:** Summary of culture methods used in OMEC sheet preparation

Ref.	Culture system	Substrate	Feeder layer	Nutrient	Air lifting	% SC	Medium	GMP	Carrier	Culture time (days)
[13]	S	dAM	3T3	10% FBS	Y	–	DMEM/F12 (1:1)	N	dAM	14–21
[14]	S	CellSeed^b^	3T3	NA	N	2.1 ± 0.9	NA	N	Carrier-free*	14
[15]	S	dAM	3T3	10% FBS/10% AS	Y	–	DMEM/F12 (1:1)	N	dAM	15–16
[16]	S	dAM	3T3	10% FBS^a^	Y	–	DMEM/F12 (1:1)^a^	N	dAM^a^	14
[17]	S	dAM	3T3	5% AS	Y	–	KGM	N	dAM	15–16
[18]	S^a^	dAM^a^	3T3^a^	U^d^	Y^a^	–	DMEM/F12 (1:1)^a^	N^a^	dAM	U^d^
[19]	S	dAM	3T3	10% FBS	Y	–	DMEM/F12 (1:1)^a^	N	dAM	> 14
[20]	S	dAM	3T3	5% FBS	N	–	SHEM	N	dAM	14–21^a^
[21]	S	dAM	3T3	5% FBS	N	–	U	N	dAM	14–21^a^
[22]	S	dAM	3T3	10% AS	N	2.0 ± 1.0	DMEM/F12 (1:1)	N	dAM	18–21
[23]	S	Fibrin^c^/dAM	3T3	4% AS	Y	–	DMEM/F12 (1:1)	N	U	NA
[24]	S	dAM	3T3	5% Serum	Y	–	KGM	N	dAM^a^	15–16
[25]	S^a^	dAM	3T3	U^d^	Y	–	DMEM/F12 (1:1)^a^	N	dAM	15–16
[26]	S	CellSeed^b^	3T3	NA	N	3.4 ± 2.06	NA	N	Carrier-free**	U^e^
[27]	S	Fibrin^c^/dAM	3T3	4% AS	Y	–	DMEM/F12 (1:1)	N	Fibrin group: carrier-free*** AM group: denuded AM	8–16 (Fibrin)/NA (dAM)
[28]	S	dAM	3T3	5% FBS	N	–	SHEM	N	dAM	14–21^a^
[29]	S^a^	dAM^a^	3T3	10%^a^ FBS/5%^b^ AS	Y	–	U^d^	Y	dAM^a^	8–9
[30]	S^a^	dAM^a^	3T3	10%^a^ FBS/%^c^ AS	Y	–	U^d^	Y	dAM^a^	8–9
[31]	E	iAM	N	AS	U	~ 12	DMEM/F12 (3:1)	Y	iAM	21
[32]	S	dAM	3T3	10% FBS/10% AS	Y	–	DMEM/F12	N	dAM	7
[33]	S	dAM	N	Serum-free	N	–	KBM-2	N	dAM	14–21
[34]	S^a^	dAM	3T3^a^	10% FBS^a^	Y	–	DMEM/F12 (1:1)^a^	N	dAM	14–21
[35]	S	BM-free	3T3	10% FBS	N	NA	DMEM/F12 (3:1)	Y	Carrier-free****	7–12
[36]	S	dAM	3T3	5% FBS	Y	–	DMEM/F12 (3:1)	N	dAM	U^f^

*AM* amniotic membrane, *AS* autologous serum, *BM-free* biomaterial-free, *dAM* denuded amniotic membrane, *iAM* intact amniotic membrane, *E* explant, *FBS* fetal bovine serum, *DMEM/F12* Dulbecco modified Eagle’s medium (DMEM) with HAM F12 mixture, *GMP* good manufacturing practice, *KGM* keratinocyte growth medium, *KBM-2* serum-free Keratinocyte Growth medium, *N* no, *NA* not available, *S* suspension, *SHEM* supplemented hormonal epithelial medium, *U* unclear, *Y* yes, *3T3* 3T3 murine fibroblasts, *%SC* percentage of transplanted stem cells

^a^According to the referenced protocol in the paper

^b^CellSeed, temperature-responsive cell-culture inserts (CellSeed Inc., Tokyo, Japan)

^c^Fibrin-coated inserts

^d^Conflicting data among the referenced studies

^e^For at least 4 days after the confluence

^f^For at least 5 days after the confluence and then air-lifted for 1 to 2 days

*Supporter

**Polyvinylidene fluoride (PVDF) ring

***Filter paper

****Support mesh

#### Medium

Dulbecco Modified Eagle’s Medium with HAM F12 mixture (DMEM/F12) was used in more than half of the studies; in ten of these, the DMEM/F12 ratio was 1:1 [13, 15, 16, 18, 19, 22, 23, 25, 27, 34], and in three of them 3:1 (Table 3) [31, 35, 36]. Other studies used supplemented hormonal epithelial medium (SHEM) [20, 28], keratinocyte growth medium (KGM) [17, 24], or serum-free keratinocyte growth medium (KBM-2) [33].

Fetal bovine serum (FBS, or FCS when referred to as “fetal calf serum”) was used in nine studies [13, 16, 19–21, 28, 34–36], five used autologous serum (AS) [17, 22, 23, 27, 31], and four used FBS and AS (Table 3) [15, 29, 30, 32]. Only one was serum-free [33]. Use of AS eliminates exposure to xenogeneic compounds contained in animal serum. One study compared use of AS with FBS and found that cell sheet morphology and expression of structural proteins were similar in both groups [17]. Preliminary in vitro work has also shown that AS promotes similar expression of putative stem cells markers in cultured OMEC sheets compared to use of FBS [31]. The two patients receiving AS feeder-free cultured OMEC sheets in this study had significant improvement in corneal epithelium integrity, pain relief, and visual acuity (VA) [31].

#### Airlifting

Fifteen studies (258 eyes) used airlifting to promote formation of a stratified epithelium (Table 3) [13, 15–19, 23–25, 27, 29, 30, 32, 34, 36]. Airlifting produced more stratification with four to nine layers compared to two to five in non-air-lifted OMEC sheets. Stratification promotes cell-cell adhesion between superficial epithelial cells via tight junction formation, which helps to prevent loss of the transplant due to blinking [49]. On the other hand, highly differentiated air-lifted sheets have lower proliferative function, which is consistent with a decrease in p63α-expressing stem cells [50].

#### Substrate

AM was the most common culture substrate (Table 3). Eighteen studies used denuded AM (epithelial layer removed) [13, 15–22, 24, 26, 29, 30, 32–34, 36], and one used intact AM [31]. Of the remaining studies, two used either denuded AM or fibrin-coated culture inserts [23, 27], two used temperature-responsive cell-culture inserts [14, 26], and one study did not employ a substrate [35].

#### Carrier

Most studies employed AM as a culture substrate and OMECs were transferred directly on the same substrate (Table 3). Two studies using temperature-responsive cell-culture inserts transferred cells on a supporter [14] or polyvinylidene fluoride membrane rings [26], which were removed after transfer to the cornea. A filter paper ring was used to transfer cell sheets grown on a fibrin substrate [27]. A support mesh was used in one study employing substrate-free culture [35].

#### Phenotype of cultured cells and presence of stem cells in culture

Immunohistochemistry and RT-PCR have shown that cultured OMECs are positive for keratin (K)3, K4, and K13 [13, 14, 17, 21, 26, 31–33], the latter is not expressed in the corneal epithelium [51]. OMECs also express markers of corneal differentiation connexin 43, laminin 5 [52, 53], and putative stem cell markers ß1-integrin, p75, p63, ABCG2, C/EBPδ [52, 54]. They do not express corneal-specific K12 and transcription factor PAX6 [22, 31]. However, heterogeneous populations of progenitor cells and mature epithelial cells in oral mucosal epithelium are similar to normal in vivo corneal epithelium; thus, its feasibility as a functional ocular surface epithelium [55, 56].

It has been shown that the presence of at least 3% stem cells (defined as ΔNp63α-positive cells) is associated with clinical success in the treatment of LSCD using CLET [57]. It is likely that the percentage of stem cells in grafted OMEC sheets also influences COMET success. Nishida et al. showed p63 expression in the basal layer of OMEC cultures used in the successful treatment of four patients with LSCD (Table 3) [14]. Analysis of putative stem cell markers (ΔNp63α, ABCG2, and C/EBPδ) in transplants have shown that OMEC and limbal cells have similar expression levels [31]. Four studies employed the colony-forming efficiency (CFE) assay to show the presence of stem cells in OMEC sheets (Table 3) [14, 22, 26, 31]. To date, any correlation between stem cell content in OMEC sheets before transplantation and clinical success using COMET remains to be investigated.

### Follow-up and clinical outcome

The shortest reported follow-up period was 1 month [35]; two studies had less than 1 year [26, 35], ten studies 1 to 2 years [13–17, 19, 22, 30, 32, 36], and nine studies between 2 and 3 years [20, 21, 23, 25, 27, 29, 31, 33, 34]. Only two studies had a follow-up time longer than 3 years [24, 28], in which the longest was 7.5 years (Table 4) [24].

**Table 4:** Clinical results, complications, and follow-up

Ref.	Complications	Stable ocular surface, ***n***/***N*** (%)	VA improvement, ***n***/***N*** (%)	Improvement in at least 2 lines of BCVA, ***n***/***N*** (%)	Mean follow-up ± SD (range) in months
[13]	Corneal epithelial defect/bacterial infection × 2	6/6 (100)	6/6 (100)	6/6 (100)	13.8 ± 2.9 (11–17)
[14]	No complications	4/4 (100)	4/4 (100)	4/4 (100)	14 (13–15)
[15]	Epithelial defect × 5	13/15 (86.7)	12/15 (80)	12/15 (80)	20 (3–34)
[16]	No complication	2/2 (100)	2/2 (100)	2/2 (100)	22.5 (19–26)
[17]	Bacterial infection × 1, epithelial defects × 4	10/10 (100)	9/10 (90)	9/10 (90)	12.6 ± 3.9 (8–19)
[18]	Bacterial infection × 1, recurrent small epithelial defects × NA	4/6 (66.7)	NA	NA	NA
[19]	Increased intraocular pressure × 1	4/4 (100)	4/4 (100)	4/4 (100)	16 (6–24)
[20]	NA	NA	4/4 (100)	4/4 (100)	31 (27–35)
[21]	Microperforation × 1, PED × 1	NA	5/5 (100)	5/5 (100)	29.6 ± 3.6 (26–34)
[22]	Corneal graft rejection × 2	5/10 (50)	5/10 (50)	3/10 (30)	18.6 (1–38)
[23]	PED × 19, stromal melting or perforation × 8, corneal infection 3 (bacterial infection × 2, recurrence of epithelial herpes simplex × 1), glaucoma × 8 (3 were new), evisceration × 2	23/40 (57.5)	23,6/40 (59)	NA ^d^	25.5 (6–54.9)
[24]	PED × 7, bacterial infection × 1, ocular hypertension × 3	NA	18/19 (95)	15/19 (79)	55 ± 17 (36–90)
[25]	Recurrence of entropion × 1, epithelial defect × 1, Symblepharon 1	2/3 (66.7)^a^	NA	NA	30 (11–50)
[26]	Symblepharon × 1, Pain and graft complication × 1, inflammation × 2, corneal graft rejection × 1, keratitis × 1, increased IOP × 1, corneal perforation × 1, Meibomian cyst × 1, pain and corneal recurrence × 1	NA^b^	17/23 (73.91) ^c^	16/23 (69.5)^c^	11.83 (NA)
[27]	Small epithelial defect × 1, PED × 10, ocular hypertension × 3	Substrate-free: 10/16 (62.5) AM: 6/16 (37.5) Total: 16/32 (50)	Substrate-free: 11/16 (68.8) AM: 7/16 (43.8) Total: 18/32 (56.3)	Substrate-free: 11/16 (68.8) AM: 7/16 (43.8) Total: 18/32 (56.3)	25.26 ± 10.8 (14.45–36.08) (substrate-free)^e^ 33.73 ± 17 (16,68–50.79) (AM)
[28]	Glaucoma × 1	6/6 (100)	6/6 (100)	6/6 (100)	36.7 + 17 (16–56)
[29]	Hepatic dysfunction × 1, drug-induced allergy × 1, PED × 16, corneal stromal melting × 2, keratitis × 1, endophthalmitis × 1, infiltration × 3, increased IOP × 4	NA	26/46 (56.52)	25/46 (54.3)	28.7 (6.2–85.6)
[30]	Epithelial defect × 3, increased IOP × 2, bacterial infection × 1	10/10 (100)	2/10 (20)	2/10 (20)	22.79 (5.6–39.7)
[31]	Central corneal epithelial defect × 1	2/2 (100)	2/2 (100)	2/2 (100)	31 (21–41)
[32]	Stromal scarring or conjunctival vascularization or stromal vascularization × 3, epithelial defect × 4	13/17 (76.5)	15/17 (88.2)	15/17 (88.2)	16 (12–18)
[33]	PED × 1, perforation × 1	15/20 (75)	14/20 (70)^d^	NA	31.9 ± 12.1 (8–50)
[34]	Epithelial defect × 3, PED × 1, bacterial keratitis × 1, increased IOP × 2, endothelial graft rejection × 4	13/14 (92.9)	14/14 (100)	14/14 (100)	28.2 ± 8.0 (14–40)
[35]	Central epithelial defect × 1, symblepharon × 1, PED × 1, primary failure × 1, recurrence of an epithelial defect × 2	6/8 (75)	5/8 (62.5)	5/8 (62.5)	9.96 ± 4.7 (2.07–15,8)^f^
[36]	Epithelial defect × 3, PED × 9, increased IOP × 2, stroma melting × 5	18/34 (52.94)	14/34 (41.17)	5/34 (14.7)	16.1 ± 5.8 (range NA)
	Total	172/243 (70.78)	225.6/331 (68.15)	172/271 (63.46)	

*n/N* number of eyes/total number of eyes, *BCVA* best-corrected visual acuity, *IOP* intraocular pressure, *NA* not available, *PED* persistent epithelial defect, *VA* visual acuity

^a^100% after repeated transplantation

^b^There was a success rate of 16/25 (64%), but it is based on a composition of criteria, not on a stable ocular surface per se

^c^It excluded from the results two patients who had serious adverse events

^d^There is no mention if visual improvement was at least of two lines

^e^Follow-up was originally given in weeks as it follows: 109.8 ± 47 weeks (substrate-free) and 146.6 ± 74.1 weeks (AM)

^f^Follow-up was originally given in days as it follows: 303 ± 144 (63–482) days

#### Success rate

Clinical success was most consistently defined in terms of a stable ocular surface. Secondary objectives reported were improved VA and best-corrected VA (BCVA). Post-graft investigations rarely included IVCM [16, 21] or IC [19]. Satake et al. used IC to show that in 2/4 eyes, the oral mucosa phenotype persisted for up to 16 months post-operatively, and in some cases the assessed epithelium displayed a mixture of oral mucosal and conjunctival cells [19].

**Fig. 2:**
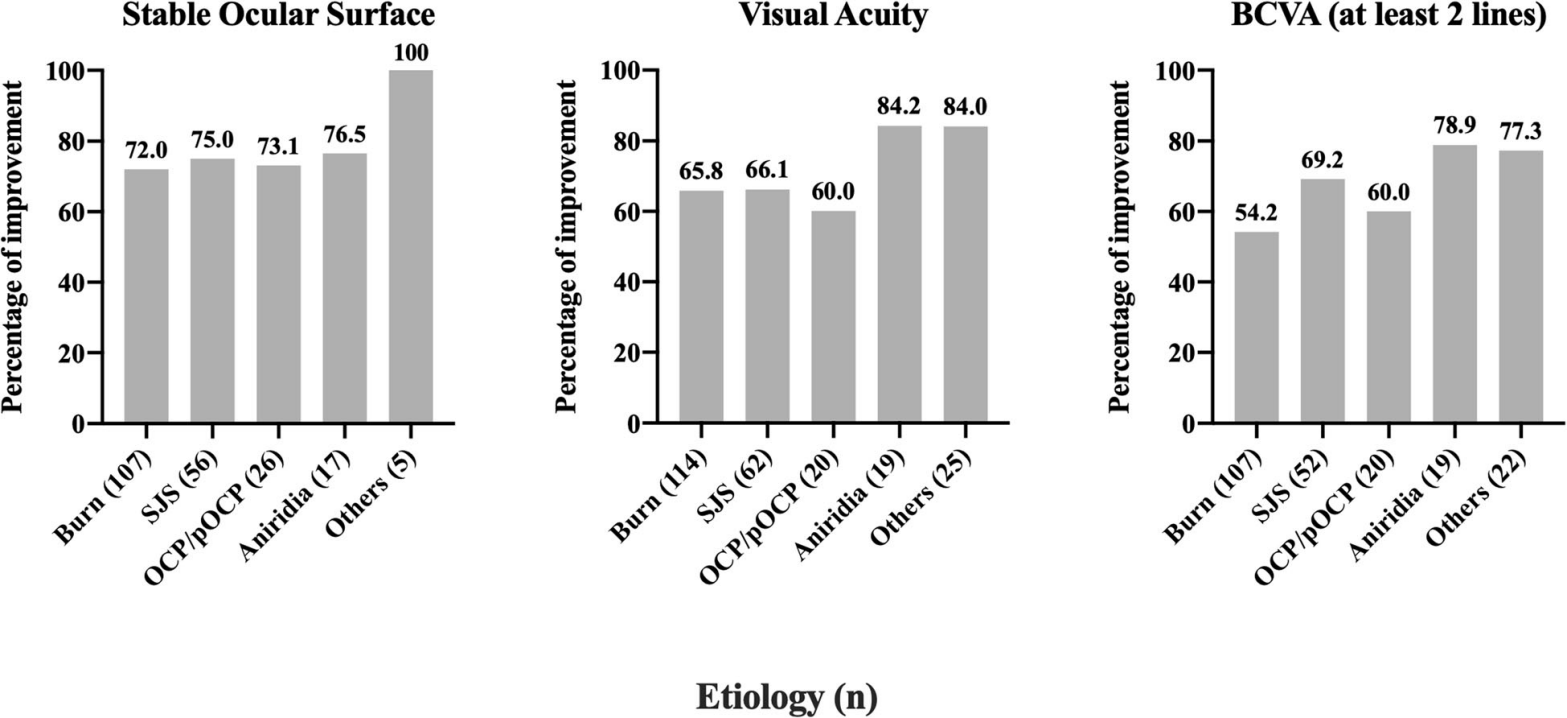
Results per etiology. OCP, ocular cicatricial pemphigoid; pOCP, pseudo-ocular cicatricial pemphigoid; SJS, Stevens-Johnson syndrome. Others: advanced pterygium, Behcet’s disease, contact lens hypoxia, contact lens hypoxia + congenital aniridia, cystinosis, drug toxicity, gelatinous drop-like dystrophy, graft-versus-host disease, hepatitis C, idiophatic, Lyell syndrome, multiple eye surgery, neuroparalytic keratitis, ocular trauma, post keratitis, radiation keratopathy, rosacea keratitis, Salzmann’s corneal degeneration, severe Groenouw dystrophy, squamous cell carcinoma, and trachoma

In total, 70.8% (172/243) of eyes receiving COMET achieved a stable ocular surface and were defined as successful (Table 4; see Fig. 2 for detailed results per etiology). This percentage is lower compared to transplantation of cultured limbal epithelial cells (LECs) (75%) [58]. Moreover, one study directly compared COMET to transplantation of allogeneic cultured limbal epithelial transplantation (ACLET) and reported 71.4% (30/42) eyes in the ACLET group achieved a stable ocular surface, versus 52.9% (18/34) eyes in the COMET group. The authors attributed the significantly higher success using ACLET to the lower incidence of post-operative PED, superior LEC proliferation and differentiation, and the ability of LECs to more readily form a stable corneal epithelium [36].

#### Visual improvement

VA improvement was reported in all but two of the studies (Fig. 2 and Table 4), and 225/331 (68.2%) eyes had some improvement. An improvement in the BCVA of at least two lines was noted in 172/271 (63.5%) eyes (data from 20 studies). The absent or incomplete description of methodology for VA/BCVA measurement prevented an accurate comparison of results between studies. VA inconsistently measured either before or after subsequent surgeries, such as PKP, was another major confounding factor.

#### Survival of oral mucosal epithelial cells after grafting

Nakamura et al. have shown that post-COMET specimens exhibit a decrease in the number of epithelial layers from 5 to 6 in successful grafts to 2 to 5 disorganized epithelial layers in unsuccessful grafts [18]. The phenotype of COMET grafts (assessed from corneal buttons retrieved after a secondary procedure, mostly PKP) was also investigated in order to characterize the differences between successful (four samples) and unsuccessful (two samples) graft phenotypes [18]. Successful cases showed the presence of K3, a marker common to oral and corneal epithelium, in all specimens; K12, a corneal-specific keratin, presented only occasional staining in one case. K4 and K13, markers of oral mucosal epithelium, were present in both successful and failed samples. In failed specimens, one presented occasional staining for K3, but both were negative for K12. MUC5AC, a conjunctival goblet cell marker [59], was present only in both failed cases and found absent in successful cases [18].

Other studies have also assessed the expression profile post-COMET, but only in successful cases. Results were similar to Nakamura et al., showing positive staining for K3, K4, K13 and negative staining for MUC5AC [16, 20, 31, 35]. Additionally, Kim et al. showed that the corneal-specific keratin, K12, was present in all four successful COMET specimens [35]. Two other studies have indicated occasional K12 staining, shown in 2/6 specimens [16, 20]. These results suggest that the epithelium post-COMET exhibits signs of both corneal-like (K12[+]) as well as oral mucosal epithelium phenotype (K13[+]). Detection of K3[+], K4[+], K12[+], K13[+], and MUC5AC[−] in clinically successful grafts shows that cultivated OMECs survive transplantation and continue to contribute to ocular surface integrity [18, 35].

However, without clear detection of cell origin (donor/host) [60–62] it is difficult to determine clearly whether cultivated OMECs were transdifferentiated into the corneal lineage or whether the presence of corneal epithelial cells represents expansion and migration of remaining corneal cells. In vivo study on rats has shown that transplanted oral mucosal cell sheets were able to survive and retain stem/progenitor cells for at least 8 weeks post-operatively, which results in the long-term success of transplantation of cultured OMECs in LSCD patients [63]. It has been suggested that restoration of a non-inflammatory environment post-operatively may be sufficient to allow repopulation of any remaining corneal cells to the ocular surface and/or resumption of normal homeostatic function by residual limbal stem cells [64].

Success of stem cell transplantation and the long-term survival of the graft in ocular surface therapy not only depends on the features of transplanted cells, but also on the surrounding microenvironment, as it provides the necessary signals required for cell maintenance and growth [48, 65]. Huang et al. speculate that transplanted OMECs might be regulated by signals originating from healthy stroma and differentiate toward the corneal phenotype, while simultaneously maintaining the oral phenotype [56]. However, identification of the key factors necessary to promote transdifferentiation of OMECs to the corneal phenotype still requires further research.

#### Post-operative complications

**Fig. 3:**
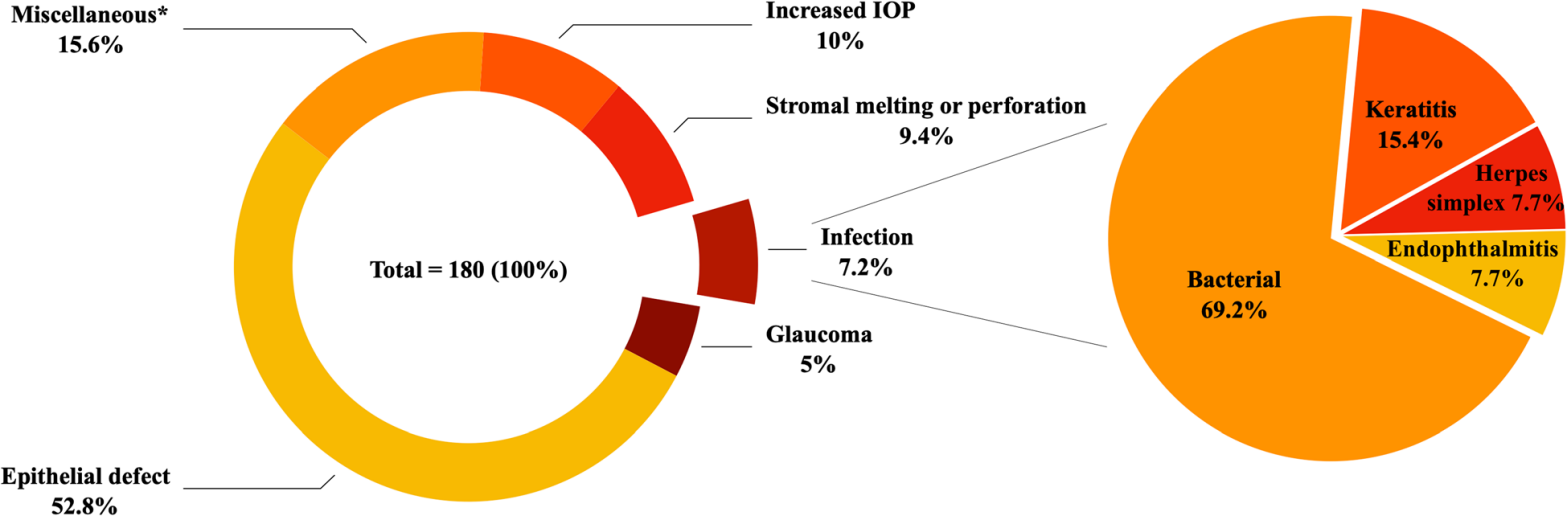
Post-operative complications. IOP intraocular pressure. *Miscellaneous (%): symblepharon (2.2), endothelial graft rejection (2.2), corneal graft rejection (1.7), stromal scarring or conjunctival/stromal vascularization (1.7), infiltration (1.7), inflammation (1.1), evisceration (1.1), entropion (0.6), primary failure (0.6), hepatic dysfunction (0.6), drug-induced allergy (0.6), pain and graft complication (0.6), Meibomian cyst (0.6), and pain and corneal recurrence (0.6)

The most common complications described following COMET were epithelial defects (52.8%; 36.1% PED), increased intraocular pressure or glaucoma (15%), stromal melting or perforation (9.4%), and infection (7.2%) (Fig. 3). For comparison, a review summarizing transplantation of cultured LECs (889 eyes) reported that the most common complications post-surgery were bleeding (8.7%), inflammation (7.5%), and blepharitis and epitheliopathy (4%) [58]. Epithelial defects making up more than half of the complications could reflect the often more serious nature of the bilateral LSCD diagnosis that demands an alternative treatment such as COMET.

Of note, there was no consensus on the definition of PED. For instance, Nakamura et al. considered epithelial defects to be persistent if they lasted for more than 4 weeks [24], while Hirayama et al. [27] defined PED occurring after 1 week following failure of conventional therapy. In a retrospective comparative study (76 eyes) a higher incidence of post-operative PED was reported in eyes receiving COMET (9/34 eyes) compared to those receiving ACLET (3/42 eyes) [36]. Several studies pointed to an association between incidence of post-operative with pre-operative PED [15, 23, 36]. It has also been shown that the transplanted epithelium exhibits decreased barrier function following COMET [19].

Baradaran-Rafii et al. suggest that PKP is inevitable in most cases of LSCD involving chemical burns due to the presence of significant corneal opacification [34]. Patients receiving PKP had improved visual function and the authors recommended performing PKP several months post-COMET to achieve the best chance of success [34].

Although most studies noted neovascularization post-transplantation [13–15, 17, 20, 21, 24, 26–28, 32–34, 36], they did not define this as a complication of the procedure. Corneal peripheral neovascularization occurred slowly in most cases, during the first post-operative year [33]. However, the central corneal area was usually spared, and neovascularization usually ceased to progress after 1 year, remaining stable thereafter [14, 33] or gradually abating with time [15, 24]. In the one retrospective study comparing ACLET and COMET, the incidence of neovascularization, corneal conjunctivalization, and improvement in symblepharon was similar between the two groups [36].

Nishida et al. pointed out that stromal vascularization observed beneath COMET transplants on the periphery of the cornea should be differentiated from subepithelial neovascularization that accompanies conjunctival ingrowth, which occurs several months post-transplantation [14]. The peripheral neovascularization seen after COMET may be caused by the lack of antiangiogenic factors, such as the soluble vascular endothelial growth factor (VEGF) receptor, fms-like tyrosine kinase-1 (sFlt-1), tissue inhibitor of metalloproteinase-3 (TIMP-3) and thrombospondin-1 (TSP-1) [28, 66, 67] or by an increase in fibroblast growth factor-2 (FGF-2) [68]. Initial in vitro work suggests that OMEC sheets produced in a culture system where 3T3 fibroblast cells are replaced with limbal niche cells as a feeder layer are less likely to induce postsurgical neovascularization [69].

#### Effect of preparation method on clinical success

We found that OMEC sheet preparation was relatively standardized; most studies used buccal tissue biopsy, DMEM/F12 culture medium, AM as a substrate and air lifting during culture. Several studies compared OMEC culture methods. The two elements that were directly compared were use of AS versus FBS in the culture medium [17] and use of substrate-free culture versus AM as a substrate [27]. Both AS and substrate-free culture have the advantage of minimizing patient exposure to potential contaminants. Clinical results so far suggest comparative or improved corneal epithelial integrity and VA with use of AS and substrate-free culture compared to the use of FBS and AM. However, larger defined comparative studies are necessary before conclusions can be drawn.

Hirayama et al. reported improved success (10/16; 62.5%) in patients receiving substrate-free OMEC sheets compared to those receiving OMEC cultured on AM (6/16; 37.5%) (Table 4) [27]. Improvement in BCVA was also superior in the substrate-free group with 11/16 (68.8%) showing improvement compared to 7/16 (43.8%). Both methods resulted in a stable ocular surface. However, graft survival was significantly improved in the carrier-free group. This may be attributed to direct contact of transplanted OMECs with stromal keratocytes and promotion of proliferation and differentiation of cells in the transplant [70].

## Conclusions

OMECs are to date the most common choice of non-limbal autologous cells in the treatment of LSCD. COMET is a promising treatment modality for LSCD, with a stable ocular surface reported in 70.8% (172/243) of LSCD eyes, and visual improvement achieved in 68.2% (225.6/331) based on published cases from the past 15 years (2004–2019).

Variation in methodologies (LSCD diagnosis, cell-culture protocols, transplantation technique, post-operative management, and measurement of VA) among the studies did not allow a precise comparative analysis of results. The use of unified tools for characterization of pre-operative status, as well as standardized assessment of outcomes would allow better comparison of studies.

## Data Availability

Not applicable.

## Abbreviations

AM: Amniotic membrane
AS: Autologous serum
ACLET: Allogeneic cultured limbal epithelial transplantation
BCVA: Best-corrected visual acuity
CFE: Colony-forming efficiency
CLAU: Conjunctival limbal autograft
CLET: Cultivated limbal epithelium transplantation
CLs: Contact lenses
COMET: Ex vivo cultivated oral mucosal epithelial cell transplantation
DMEM/F12: Dulbecco Modified Eagle’s Medium (DMEM) with HAM F12 mixture
FBS: Fetal bovine serum
FCS: Fetal calf serum
GCs: Goblet cells
GMP: Good manufacturing practice
KBM-2: Serum-free keratinocyte growth medium
KGM: Keratinocyte growth medium
IC: Impression cytology
IVCM: In vivo confocal microscopy
LECs: Limbal epithelial cells
LESCs: Limbal epithelial stem cells
LSCD: Limbal stem cell deficiency
OCP: Ocular cicatricial pemphigoid
OMECs: Oral mucosal epithelial cells
pOCP: Pseudo-ocular cicatricial pemphigoid
PED: Persistent epithelial defect
PKP: Penetrating keratoplasty
sFlt-1: fms-like tyrosine kinase-1
SHEM: Supplemented hormonal epithelial medium
SJS: Stevens-Johnson syndrome
TIMP-3: Tissue inhibitor of metalloproteinase-3
TSP-1: Thrombospondin-1
VA: Visual acuity
VEGF: Vascular endothelial growth factor
